# A new modular mechanism that allows full detachability and cleaning of steerable laparoscopic instruments

**DOI:** 10.1007/s00464-019-06849-0

**Published:** 2019-05-29

**Authors:** Sem F. Hardon, Frank Schilder, Jaap Bonjer, Jenny Dankelman, Tim Horeman

**Affiliations:** 1 Department of Surgery, VU University Medical Center, Amsterdam, The Netherlands; 2 Department of BioMechanical Engineering, Delft University of Technology, Mekelweg 2, 2628 CD Delft, The Netherlands

**Keywords:** Advanced laparoscopic surgery, Robot surgery, Instrument steering, SATA technology, Reusable vs disposable

## Abstract

**Background:**

Ever since the introduction of laparoscopic surgery, researchers have been trying to add steerability to instruments to allow the surgeon to operate with better reachability and less tissue interaction force. Traditional solutions to introduce this often use a combination of springs, cables, pulleys, and guiding structures, resulting in instruments that cannot be properly cleaned and thus are very costly to manufacture and maintain. The aim of the study is to develop a novel affordable, sustainable, cableless, and fully steerable laparoscopic grasper, and to test its ease of assembly, disassembly, and use.

**Methods:**

A set of requirements was defined to ensure that the instrument can be handled efficiently at the sterilization unit and in the operating room. Based on these, a multisteerable, cableless 5 mm laparoscopic instrument that operates based on shaft rotations was developed. To test its assembly and disassembly, ten participants were asked to fully dismantle the instrument and reassemble it a total of 60 times. In addition, ten medical students were asked to use the grasper in the ForceSense box-trainer system on a newly developed 3D pick-and-place task, to determine the control effort based on learning curves of steering errors, task time, instrument path length, and maximum tissue interaction force.

**Results:**

All important design requirements were met. The recorded data indicates that ten engineering students were able to fully dismantle and reassemble the instrument shaft in 12 s (SD7) and 65 s (SD43) seconds at the sixth attempt. The learning-curve data indicates that three attempts were needed before the ten medical students started to use all steering functions. At the sixth attempt, on average only 1.25 (SD0.7) steering errors were made. The steepest slope in the learning curves for steering errors, path length, and task time was experienced during the first three attempts. In respect of the interaction force, no learning effect was observed.

**Conclusion:**

The multi-DOF (degree of freedom) cableless grasper can be assembled and disassembled for cleaning and sterilization within an acceptable time frame. The handle interface proved to be intuitive enough for novices to conduct a complex 3D pick-and-place task in a training setting.

**Electronic supplementary material:**

The online version of this article (10.1007/s00464-019-06849-0) contains supplementary material, which is available to authorized users.

## Advanced and robotic-assisted laparoscopy

**Fig. 1:**
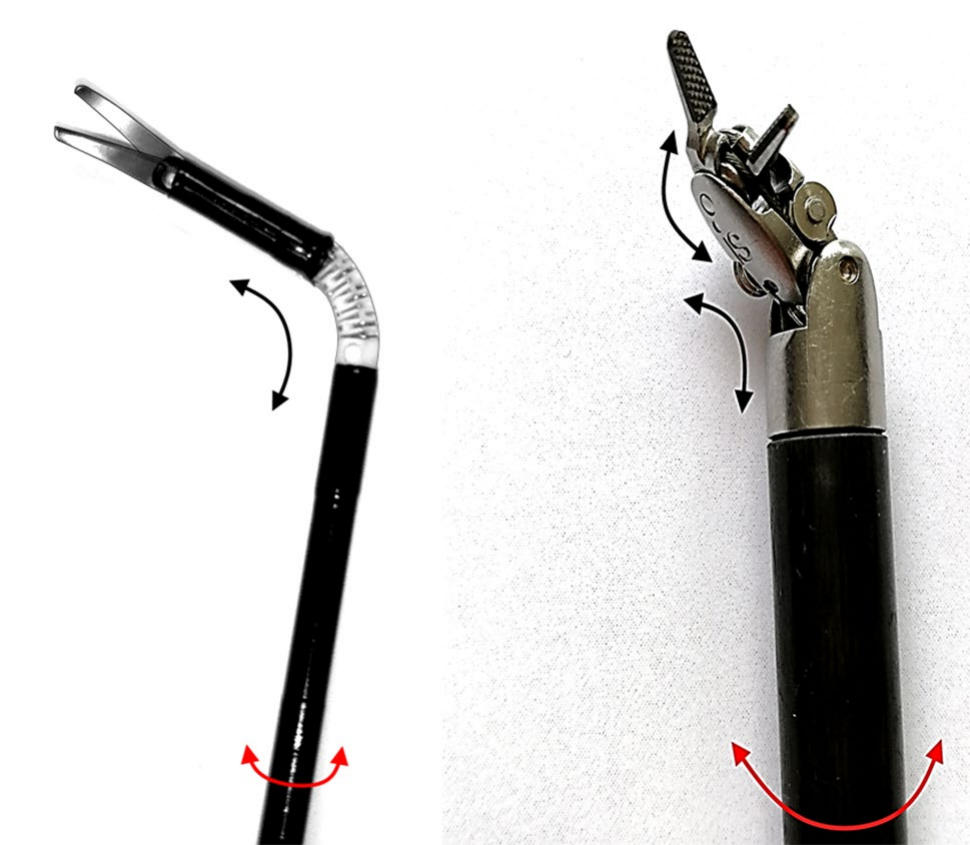
Two examples of tip steering. Left, by the tensioning and release of spring-loaded cables within a continuous deformable flexible element between tip and shaft. Right, by cable-driven stiff element articulation

Laparoscopy, a form of minimally invasive surgery (MIS) performed in the abdominal region, was introduced to overcome intense tissue trauma, large cosmetic scars, and long hospital stays [1–5]. Since laparoscopy is performed through small incisions in the abdominal wall, long slender instruments (Fig. 1) are needed to manipulate, cut, and suture tissues inside the CO_2_-inflated abdomen. As procedures become more complex, an increasing number of surgeons move from conventional laparoscopy to robotic surgery or start to use advanced steerable, hand-held laparoscopic instruments. When a robotic master–slave system is placed between the surgeon and the instruments, the ergonomics, dexterity, precision, and accuracy improve as those systems are able to scale and filter undesirable movements (e.g. tremors) [6–8]. Despite these advantages, the high fixed and recurring costs of the robotic system and the disposable steering mechanics currently in use hamper the introduction of robotic surgery and steerable, hand-held instruments in less wealthy hospitals [6, 9].

## Steering principles

Ever since the introduction of laparoscopic surgery, researchers and manufacturers have been trying to add steerability to the tip of the instrument in order to allow the surgeon to operate with improved reachability, improved beak-tissue alignment and less tissue interaction force [10, 11]. Steering of the tip can be accomplished either using continuous deformable elements between shaft and tip, in combination with either axial tip rotation driven by the inner rod or shaft plus tip rotation driven by the outer shaft (Fig. 1, left), or using multiple hinge linkages in combination with either axial tip rotation driven by the inner rod or shaft and tip rotation driven by the outer shaft (Fig. 1, right).

## Steering with compliant components

Traditional solutions often use a combination of spring-like elements, which are either compressed or released by a set of four or more cables evenly distributed in or around the spring surface [10, 12]. Although high tip-positioning accuracy can be accomplished in this way, these mechanisms often require a significant bend radius to remain compliant since the configuration of the instrument depends on the forces acting on the tip. When more tangential and axial stiffness is needed, exoskeletons built out of interconnected rings that act like hinges can be added [13–16].

## Steering with stiff components

One commonly used method to create stiffer and more controllable steerable instruments is the use of traditional rotating hinges between the segments, as seen in the Da Vinci Surgical System (Surgical Intuitive) [17]. Alternatives that have been implemented and tested successfully in endoscopic instruments are the belted rolling-link mechanism by Herder et al. [18] and the rolling-gear mechanism by Jelínek et al. [19]. These hinge mechanisms were developed in an attempt to minimize friction, while at the same time improving stability. Other hinge principles that rely on sliding surfaces, as described in the review by Jelínek et al., are less common in steerable instruments that require accurate control.

## Actuation principles

Although articulating instruments are often referred to as “steerable”, it remains unclear how these are actually used. Depending on the specific surgical action required, an instrument used for tip articulation can be either continuous or intermitted. A good example of a continuous steerable instrument is the FlexDex needle driver (Alphatron Surgical) described by Awtar et al. [20, 21]. Suturing with a curved needle requires a smooth and coupled rotational motion that follows the line of the needle, so it seems relevant to copy the wrist motion of the hand from outside into the abdomen. During other less complex surgical actions, such as cutting a damaged meniscus during a knee arthroscopy, it seems relevant to allow the surgeon to use the steering function only to adjust the tip configuration when following the rim of the meniscus before loose parts are cut and removed [22, 23]. In many other common situations, there is a need to line up the beak of the instrument with the tissue to be manipulated (e.g. when stretching soft tissue during dissection and when using clip appliers). Therefore, the purpose for which the surgical instrument is used should be considered when choosing the method of steering, since the complexity of actuation influences both the mental burden on the user and the manufacturing cost.

## Complexity of steerable instruments

While almost all recently developed conventional reusable (e.g. non-steerable) laparoscopic instruments are designed to be taken apart for cleaning, the state of the art in steerable instruments for advanced laparoscopic (e.g. robotic) surgery are disposable and cannot be dismantled since they contain many hidden components. Components that are often hidden inside the instrument shaft are cables, springs, pulleys, and support structures needed to translate finger and hand movements from the handle into translations and rotations of the instrument tip [17].

Particularly in the field of minimally invasive robotic surgery, the conventional approach used to create steerability results in surgical instruments that are nearly impossible to inspect in the OR or central sterile services department (CSSD) and require expensive equipment for cleaning and maintenance. As a result, most steerable laparoscopic instruments are disposable and therefore a potential financial burden for those wishing to undertake robotic surgery at financially unstable hospitals not equipped with the most advanced cleaning systems in the CSSD. Winter et al. found that, to gain acceptance of a technical innovation at a low-resource hospital, a different design approach is often required [24]. This can be accomplished by developing intuitive and maintenance-friendly instrumentation that can be used by surgeons working in poorly resourced hospitals with basic CSSDs. We have, therefore, used a “bare-minimum design” methodology, with a strong focus on component interaction analysis and adding functions to standard components [22], in combination with a stepwise development and evaluation plan that involves all key users who come into contact with the innovation to create a new steering mechanism.

## SATA articulation technology

In a previous study by Horeman et al. [22, 23], a single-DOF shaft-actuated tip articulation (SATA) mechanism was developed and used to build a reusable steerable punch for meniscectomy. Since this operates without cables, the instrument’s inner space remains relatively open, leaving room for extra components required for additional tip functions such as electrosurgery, optics, or mechanical movement to initiate cutting or grasping. Unlike the hinges in currently available instruments such as EndoWrist, AutoSuture, FlexDex and Endo Grasp, the SATA mechanism can be disassembled for cleaning and has a smaller bend radius, making it ideal for endoscopic procedures performed in small spaces like joints or hollow organs. Due to the potential of the cableless steering approach, the aim of this study was to develop a fully detachable and cleanable 5 mm, two degrees of freedom (2DOF) articulating grasper for laparoscopic surgery.

## Requirements

The following requirements for a reusable steerable laparoscopic instrument need to be met to address known clinical functioning prerequisites.

- To fit most standard trocars for 5 mm instruments, the outer diameter should be less than 5.3 mm.
- Build out of autoclavable components [22].
- Can be disassembled for cleaning [22, 23].
- No hidden areas in the components entering the abdomen that cannot be inspected or cleaned [22, 23].
- Reusable for a minimum of 30 surgical procedures [22, 23].
- The device can be assembled by a single person in less than 110 s [22].
- The device can be disassembled by a single person in less than 110 s [22].
- Jaw and pitch degree of freedom greater than 60 degrees to mimic hand motions [25]
- Jaw opening angle greater than 45 degrees [26].
- Grasper jaws capable of grasping tissue with at least 56 Nmm of pinch torque when device is not articulated [27].
- Minimal gas leakage through the instrument shaft to maintain pneumoperitoneum.
- Similar as conventional laparoscopic instruments, shaft + tip length should be no less than 330 mm [27].

## Methods

### Mechanical design

#### Instrument assembly and actuation

**Fig. 2:**
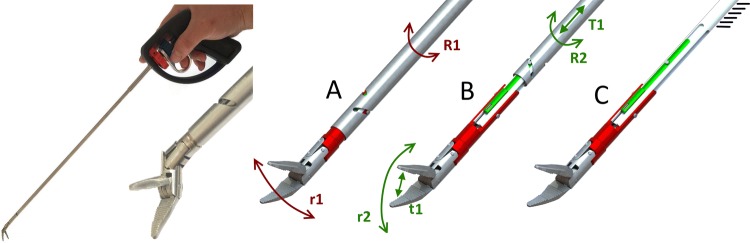
Left, the SATA 2DOF instrument and tip. Right, principles of tip actuation. **A** Sideways articulation (**r1**) is accomplished by rotation of the outer tube (**R1**), which drives the red sliders. **B** Beak rotation (**r2**) and closure or opening (**t1**) are accomplished by rotation (**R2**) or translation (**T1**) of the inner tube, which drives the Green sliders. **C** The inner rod fixed to the handle prevents rotation of all the sliders, while allowing translation

**Fig. 3:**
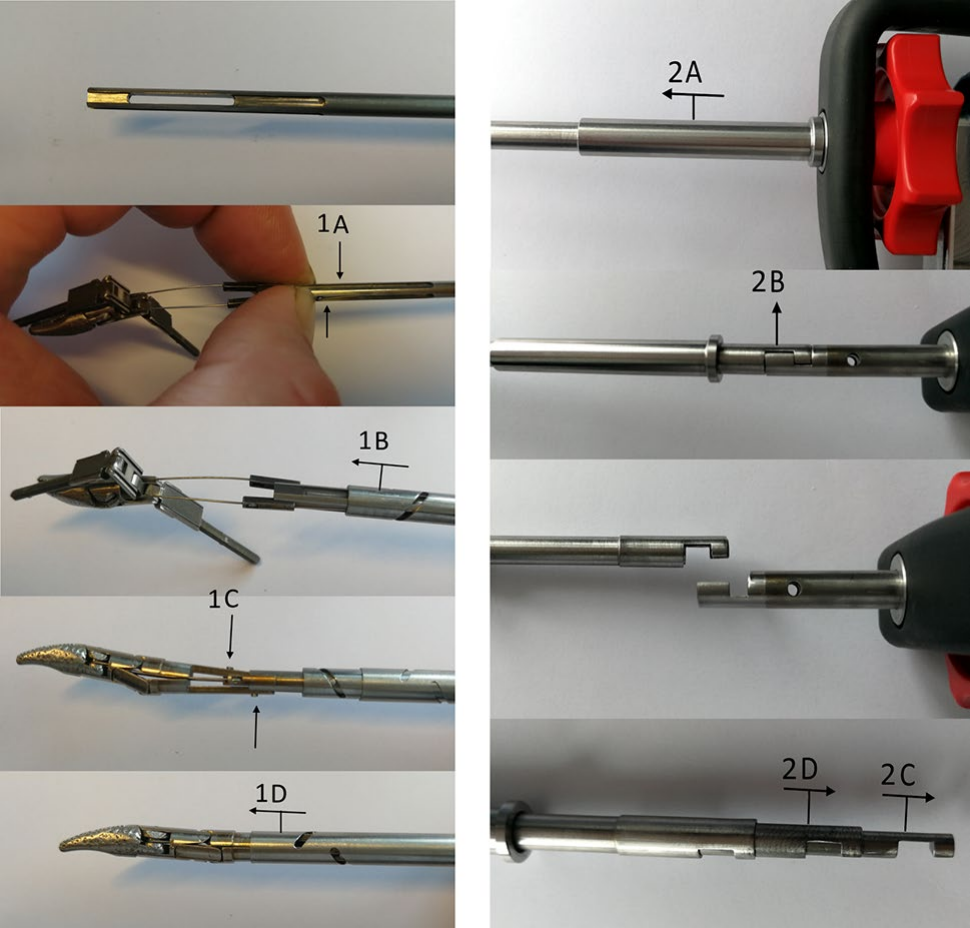
Critical steps in the shaft assembly (left) and disassembly (right) procedure. To assemble the shaft (left, top to bottom), the first set of sliders is pressed into the opening in the rod (1A) so that the smallest inner tube can slide over the first set of sliders until both pins click into the helix fissures (1B). The rod is then pushed forward towards the tip to hold the first set of sliders in place until the second set of sliders falls neatly into the distal cutout in the rod (1C). Then the outer tube is moved distally until the pins of the second slider set click into the fissures. In a final step, the inner rod is pushed further towards the tip to secure the second set of sliders as well (1B). The shaft assembly is now ready to be locked to the handle or control box. To detach the “puzzle-piece” connection (right, top to bottom), the locking tube is moved sideways (2A) to expose the coupling and inspection holes in the tubes and rod. When all the holes in the tubes are visually aligned (2B), so are all the puzzle pieces. They can now be disconnected in a single motion. Once unlocked, the tubes and rod can be removed from the shaft assembly (2C, 2D)

A functional prototype was built for testing (Fig. 2, left). For sideways articulation (i.e. tip yaw), this uses the SATA actuated three-point hinge mechanism as previously described by Horeman et al. [22]. The sliders that steer this mechanism are modified to guide an additional pair of Nitinol wires connected to both beaks and so facilitate their pitching (Fig. 2A). To rotate the beaks, two additional sliders are added to the existing pair of beaks (Fig. 2B). The sliders, four in total (i.e. two pairs), are actuated by two independent tubes, each actuated by a separate steering wheel. An inner rod with four elongated cutouts at the distal end (i.e. tip side) is used to prevent axial rotation of the sliders when actuated by the tubes’ beaks (Fig. 2C). The only connection between the fully steerable tip and handle is established using three cylindrical tubes. To assemble the shaft, the first set of sliders is pressed into the opening in the rod (Fig. 3, 1a) so that the smallest inner tube can slide over the first set of sliders until both pins click into the helix fissures. The rod is then pushed forward towards the tip to hold the first set of sliders in place until the second set of sliders falls neatly into the distal cutout in the rod (Fig. 3, 1C). Then, the outer tube is moved distally until the pins of the second slider set click into the fissures. In a final step, the inner rod is pushed further towards the tip to secure the second set of sliders as well. The shaft assembly is now ready to be locked to the handle or control box.

#### “Puzzle-piece” shaft connection

To couple the shaft to the handle, a new type of connection has been developed. This “shape-fit” or “puzzle-piece” connection allows the assembled shaft to be locked onto the handle in a single action, thanks to the self-aligning ability of the six puzzle pieces. Figure 3, right, shows this process in chronological order. First, the puzzle-piece coupling and inspection holes are exposed when the 40 mm locking tube is moved sideways (Fig. 3, 2A). The puzzle-piece connection is then unhooked in a single motion (Fig. 3, 2B). Finally, the inner tubes can be pulled out of the assembly to release the tip (Fig. 3, 2C). Visual inspection holes are included for the user to see when all parts are aligned for decoupling. Only when the user is able to look right through all the holes in line can the shaft be detached.

#### User interface and control

**Fig. 4:**
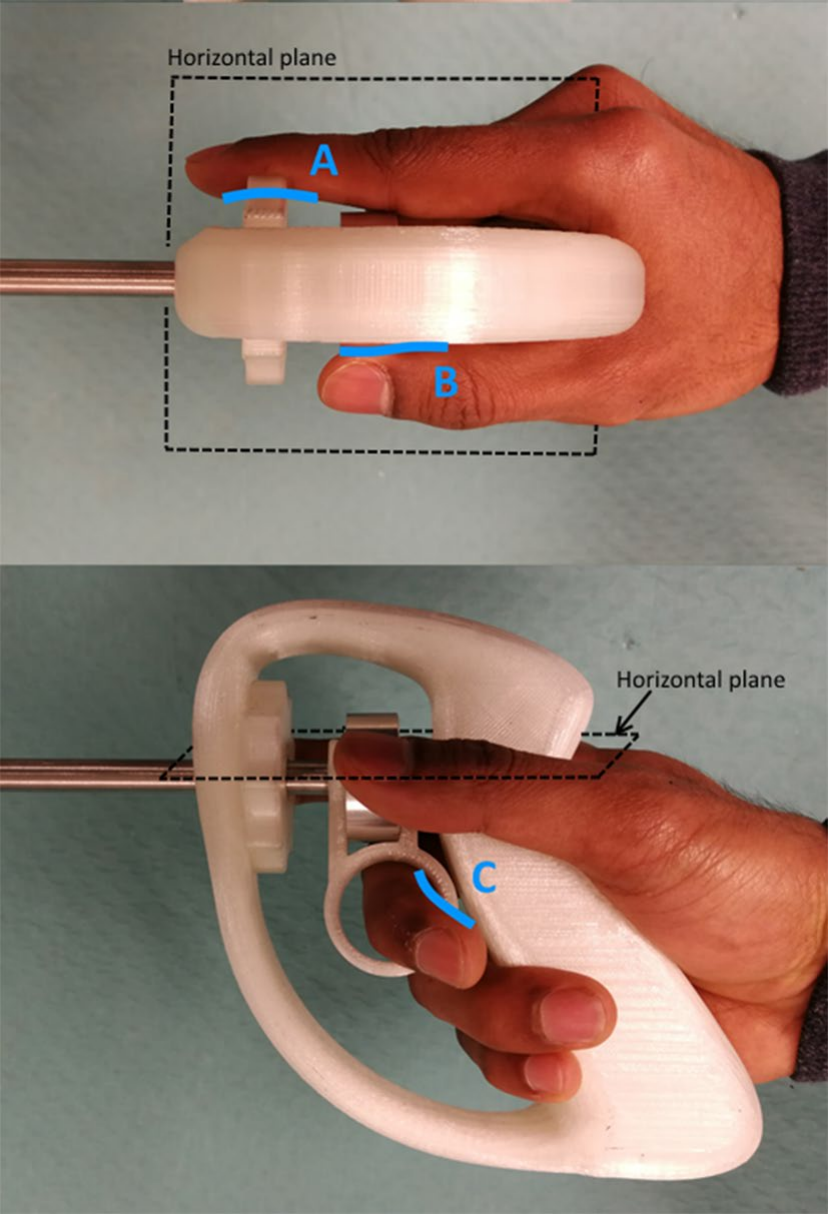
A handle design for hands of different sizes. The wheel actuation motion and position have been chosen in such a way that they and the reachability of the wheel (**A, B**) are not dependent on the length of the user’s finger, since both wheels are rotated with stretched fingers. As both are actuated in the same horizontal plane, the middle finger can be used freely to trigger the ring lever that actuates beak opening and closing (**C**). The rest of the fingers are used to establish a firm grip

**Fig. 5:**
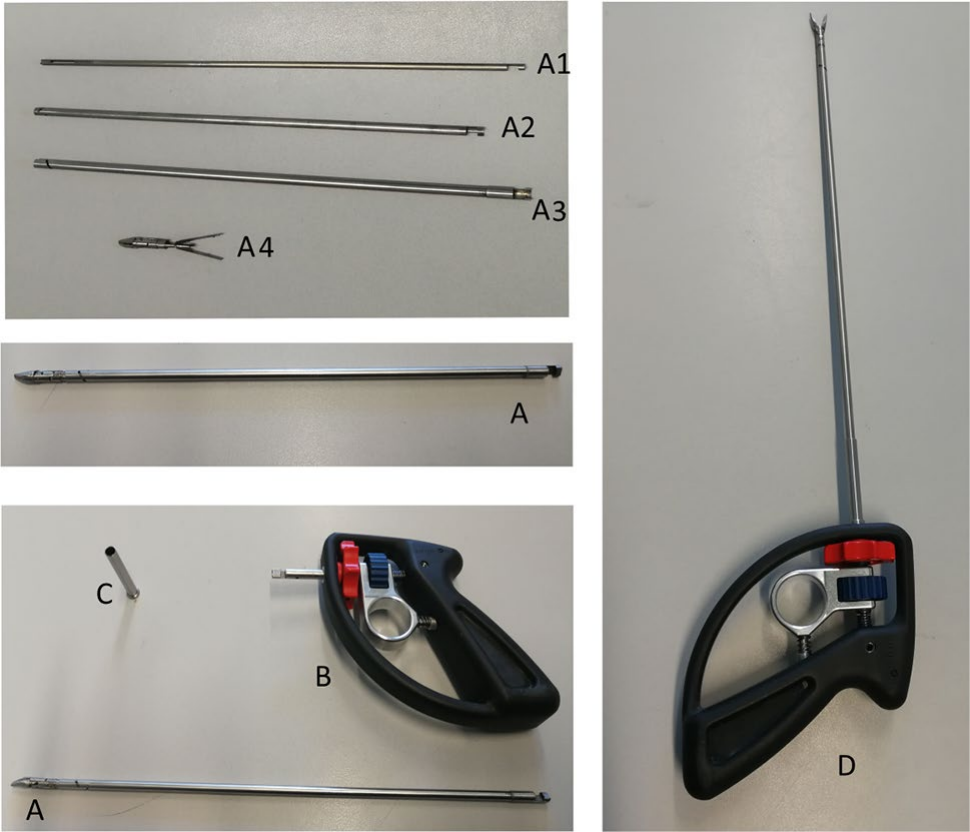
Assembly and disassembly of the detachable multi-DOF grasper. Top left, the shaft contains the inner rod (A1) and the tubes (A2, A3), and at the end is the tip with sliders (A4). Middle left, the assembled shaft. Bottom left, the shaft (A) and handle (B) are connected and secured by sliding the coupling tube (C) over the connection

**Fig. 6:**
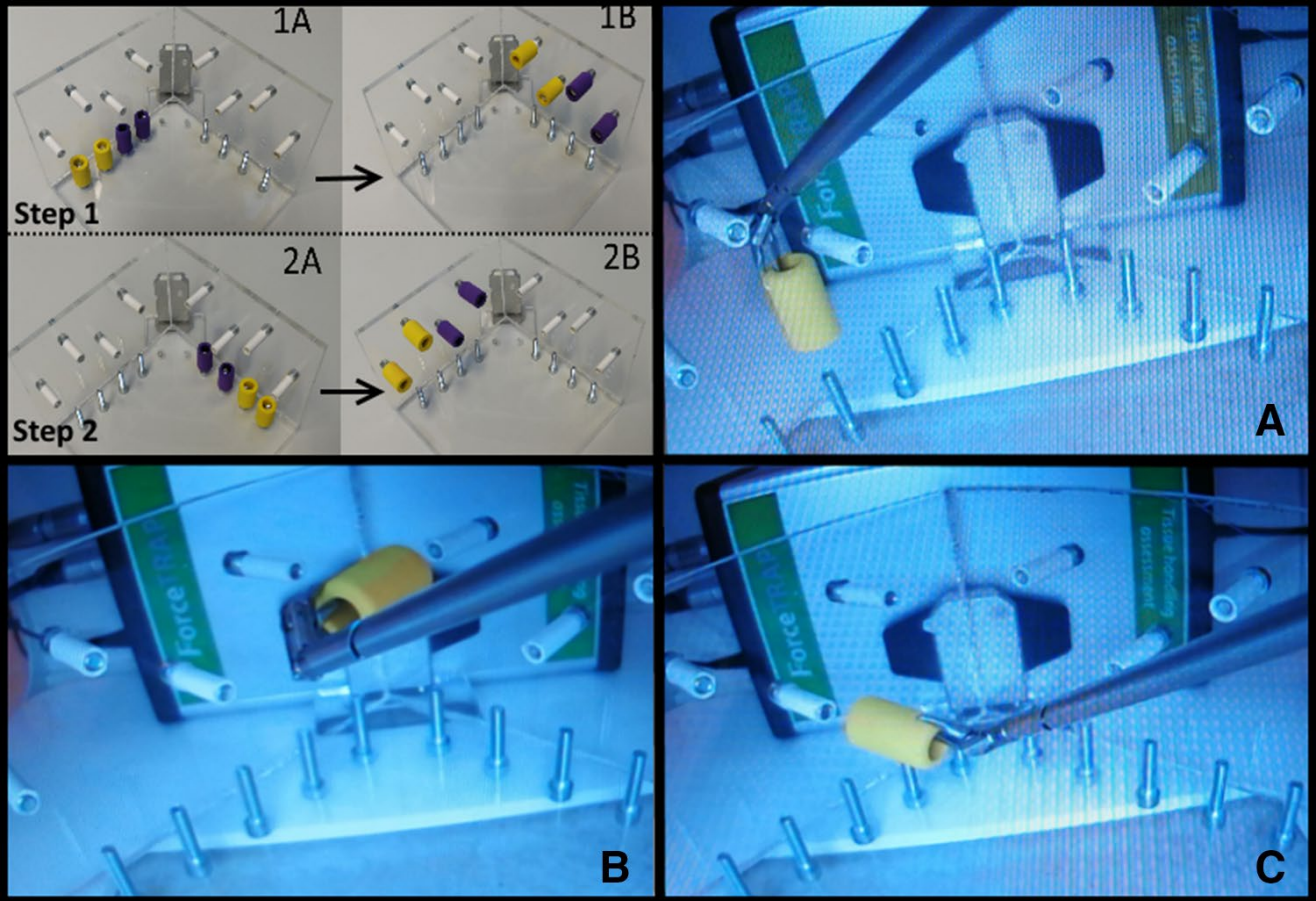
3D training in ForceSense. Top left, steps needed to complete a customized 3D pick-and-place training task. 1A and 1B show the locations of the colored elastic tubes before and after the first step. 2A and 2B show their locations of the colored elastic tubes before and after the second step. Top right, bottom left and bottom right, pictures taken during a subject’s final attempt at the task and showing the instrument tip configuration needed to pull the yellow tube cleanly from a pin on the horizontal plane (A) and to slip it over a pin on the the right-side (B) or left-side (C). As the fit is tight, smooth placement is only possible with perfect alignment

To control the instruments, we aimed for a pistol grip with hand-size independent steering-wheel actuation. This pistol grip is equipped with two steering wheels, for pitching and yawing, and one actuation lever for pinching. Looking at the human hand, we have concluded that the ring finger is best used as the “trigger finger” to actuate both articulation wheels and the pinching lever, leaving the index finger and thumb free to rotate the two wheels (Fig. 5). The difference in size between the front and back wheels (A, B) facilitates accurate independent finger-length control of both without the base of the index finger interfering with the back wheel. As both interactions (A, B) occur roughly in the same plane, the lever-ring finger interaction does not interfere with steering and the design of the handle remains very simple. Once the basics of the design were determined, the model shown in Fig. 6 was used to tweak the dimensions based on feedback from ten students. This resulted in the model as presented in Fig. 4.

### Assembly experiment

To gain knowledge about the design’s potential in terms of usability in the OR and CSSD, it was important to test whether the instrument can be assembled and disassembled within a reasonable time frame and without damaging any of the parts. In the first part of this experiment, ten Biomechanical Engineering students (eight male, two female) were asked to assemble the tip-shaft configuration (Fig. 5, A) and then completely disassemble it (Fig. 5, A1–A4) six times in all. In the second part of the experiment, the same subjects were asked to connect the shaft assembly to the handle (Fig. 5, D) and to test the DOFs, then disconnect the shaft from the handle (Fig. 5, A–C). As instruction, the step-by-step assembly and disassembly procedure was first demonstrated once by the supervisor. The subjects then practiced assembly and disassembly twice with feedback from the supervisor. Following this practice, the time taken to complete each of the six unaided attempts at assembly and disassembly was measured. All of the subjects had previously used the assembled instrument in a training environment but had no prior experience of its assembly or disassembly.

### Functional testing

To investigate whether the instrument’s design and actuation are functionally intuitive, ten right-handed medical students in their first and second years (five male, five female) with no prior experience of laparoscopic surgery were asked to perform a specially designed 3D pick-and-place task (Fig. 6, top left) based on a fundamental laparoscopic surgery (FLS) and fundamental robotic surgery (FRS) training task [28, 29]. Different from existing training tasks, only adequate alignment facilitates smooth placement as the fit between the elastic tubes and pins on the vertical planes is tight. This task can, therefore, only be completed correctly if subjects use the steering functions of the instrument. Figure 6, bottom right, shows the required tip-and-beak articulation for adequate alignment of the beaks. A training box equipped with force and motion measurement capabilities (ForceSense v3.0, MediShield BV, Delft, the Netherlands,) was used to identify the learning curves for the most discriminating motion and force parameters (e.g. maximum force, path length, and task time) [30, 31]. In addition, the trials were video recorded and analyzed to determine the number of faulty steering actions. A faulty steering action was defined as a visual steering action in the wrong direction followed by an immediate correction in opposite direction. Only pitch and yaw articulation actions were considered. Steering errors were included from the moment the first tube was removed from the vertical pin. As the experiments were executed on a standard box trainer with mechanical instruments that are not powered by electricity nor have energy stored inside IRB approval was not required. This study was conducted blind at Amsterdam UMC, with no participation by the team that developed the technology.

### Task procedure in ForceSense

**Fig. 7:**
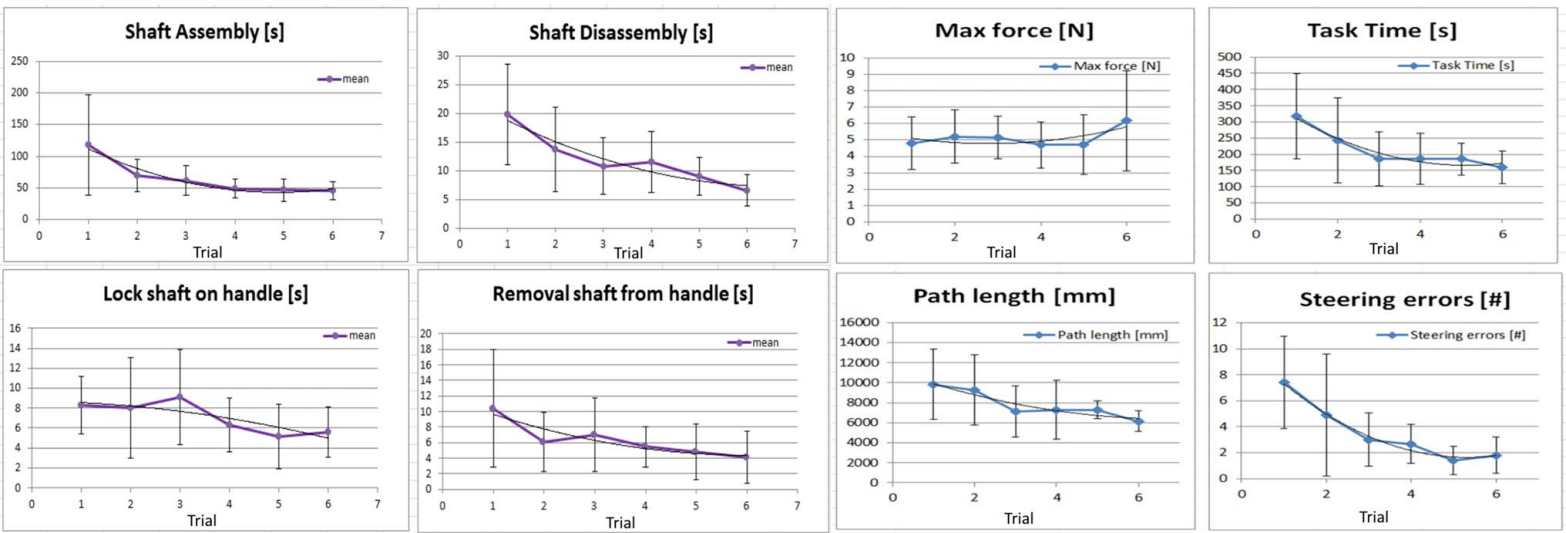
Left, learning curves for the four different assembly and disassembly tasks. Right, learning curves for the maximum force, task time, path length, and steering error parameters. Second-order polynomial trend lines have been added to indicate learning effects

Prior to the exercise, the subjects were instructed and shown how to hold the instrument in order to control the pitch motion wheel with their index finger, the yaw motion wheel with their thumb, and the pinching motion with their ring finger. This instruction was done outside the training box. Finally, as shown in the top left photo in Fig. 7, the instrument was placed installed in the training box and the subjects were asked to bring the two pairs of colored tubes from position 1A to 1B (step 1) and then from 2A to 2B (step 2). This task was performed six times.

### Testing the functional requirements

As there is a relationship between the beak opening and the pitch and yaw angles, the maximum allowable pitch and yaw angles are determined with a closed beak. To determine the pinch torque, the force is measured at the middle of each beak part when the tip opening angle is 45 degrees. In a manner similar to that described previously by Horeman et al. [22], two 0.4 mm cables are attached to each of the beaks. The first of these, connected at one end to a screw in a tabletop, runs vertically towards the tip beneath the table and is connected to the first beak part. The second cable runs vertically from the second beak part to a mass of 1 kg. As there are no pulleys involved, the force acting on the beak is defined only by the mass hanging from the cable.

### Statistics

Statistical analyses were performed using IBM SPSS Statistics (v22, IBM Corp., NY, USA). A Shapiro–Wilk test was conducted to indicate the normal distribution of the data (*p* > 0.05). To determine any difference between the first and last training task, an *F* test was used to test the null hypothesis that the variances of the populations are equal (p > 0.05). In the case of equal variance, a double-sided paired Student’s *t* test was used to test the null hypothesis that the means of the two populations are equal. The statistical significance was set at *p* < 0.05.

## Results

### General design requirements

A detachable instrument was built with an outer tube diameter of 5 mm, leaving space for an isolation tube with a maximum wall thickness of 0.15 mm. The total length of the shaft is 328 mm, including the tip, which is similar to that of laparoscopic instruments currently in use. All tip components are made of Nitinol or 316 stainless steel and are therefore autoclavable. Although designed to be injection-molded if mass-produced, the handle for the first experiments was printed, using a medical-grade plastic in order to be autoclavable. Disassembly for cleaning is possible without leaving hidden areas in the components entering the abdomen that cannot be inspected or cleaned. As the tolerances between it and the tube are less than 0.1 mm, gas leakage through the instrument shaft is negligible and so pneumoperitoneum is maintained.

### Functional requirements

Measuring the jaw angles shows an articulation range of 159 degrees (left 82, right 77). Measuring the pitch angle shows an articulation range of 110 degrees (up 55, right 55). The maximum tip opening measures between 52 and 60 degrees, slightly depending on the jaw angle. Force measurements show that the user can close the loaded tip (10 N) at least ten times without causing any visual damage to the components.

### User requirements: assembly experiment

All data was normally distributed. The *F* test indicated no significant population difference between the first and last attempts at the shaft assembly task, the disassembly task, and the removal of shaft from handle task (*p* < 0.001, *p* = 0.004, and *p* = 0.02). Learning curves were established for the shaft assembly and disassembly experiments (*p* = 0.02, *p* = 0.005). In total, the ten subjects were able to fully assemble and disassemble the instrument 60 times without damaging the components. Figure 7, left, shows the results of the experiments. Within the first phase, the shortest shaft assembly time was 27 s, the longest 308 s. The average assembly time over all trials was 65 s (SD43). Disassembly of the shaft took 12 s (SD7) on average, with a minimum and maximum of 3 s and 39 s respectively. In the second phase of the study, it took the subjects an average of 6 s (SD5), with a minimum and maximum of 1 s and 24 s, respectively, to connect the assembled shaft to the handle and to test the DOFs. Taking the assembled shaft from the handle took 7 s (SD4) on average, with a minimum and a maximum of 1 s and 18 s, respectively.

### User requirements: functional testing

Animation 1 shows one of the participants executing the last trial on the 3D training task in the ForceSense Box-trainer. All data were normally distributed. The F test indicated no significant population difference between the first and last tests of the time, path length, maximum force, and steering errors (*p* < 0.01, *p* = 0.03, *p* = 0.04, and *p* = 0.001). Learning curves were established for the maximum force, task time, path length, and steering errors (*p* = 0.01, *p* = 0.03, and *p* = 0.01). Of the 60 videos recorded, two were not saved and analyzed due to network problems that prevented their proper uploading. Observing the video data shows that 60% of the subjects started to use both steering functions efficiently at the first attempt. The remaining 40% needed a maximum of two more attempts before both steering functions were fully understood. The learning curves of each parameter are presented in Fig. 7, right. Between the first and last attempts at use, the objective ForceSense learning-curve data reveals average reductions in task time, path length, and steering errors of 53, 37, and 80%, respectively. However, the average maximum force increased by 20%.

## Discussion

A fully functional, detachable, cableless 5 mm laparoscopic instrument was created with two additional degrees of freedom at the tip. This can be assembled and disassembled for cleaning and sterilization within an acceptable time frame and using low-tech cleaning methods, and also meets the requirements set. When adding an insulation tube 0.15 mm thick to enable monopolar electrosurgery, the total diameter becomes 5.3 mm—thin enough for all standard 5 mm trocars currently on the market. Although the “puzzle-piece” connection proved efficient for coupling the shaft to an actuation handle, it is likely that robotic surgery will benefit even more from this device as it allows for replacement of disposable instruments with more financially attractive reusable modular ones.

### Assembly experiment

The average assembly and disassembly time 65 s and 12 s are shorter than the required 110 s. Since 110 s was the largest observed (dis)assembly time during an inventory conducted at 5 large Dutch hospitals [22], an average sterilization department with skilled personnel should be able to process this kind of modular instruments. The shaft-assembly tests show the largest data variation in the first attempt at those actions which require performance of the task in a specific order, plus knowledge of the interacting components within the system. During the task “lock shaft to handle,” all the moving parts can be followed visually until the connection is made. This task, therefore, requires less haptic information or knowledge of component interaction within the system, resulting in a low and constant task time during the experiment. For Phase 1, the assembly and disassembly tasks containing the most steps, a large variation is observed only between the first and second attempts. This indicates that, by the second attempt, subjects have successfully translated the theoretical knowledge into a practical approach they can use to perform the task efficiently. Thereafter, the main developments observed were improvement of technical skills and additional use of haptic information during slider positioning and the alignment of parts, further reducing the parameters in the learning curves.

### Functional testing

The handle interface proved to be intuitive enough for novices to start using the actuation wheels within seconds. On average, four to five attempts were needed to adapt to the interface. This is confirmed by Fig. 7, right, which indicates that the learning curve stabilizes at about fifth attempt, and is also in line with earlier studies involving similar tasks undertaken with conventional graspers [32, 33], demonstrating that participants’ technical skills improved most clearly in the four to five attempts. All this suggests that, although the instrument has more functions to be actuated compared with conventional ones, it does not require more training to learn to control those additional functions in this study. From dedicated studies it is known that robotic surgery requires a distinct learning curve different from laparoscopic and open surgery [34]. As this might be similar for steerable instruments a comparison between steerable hand-held instruments and robotic instruments should be conducted on validated FRS tasks to identify differences in learning curves.

### Socioeconomic value

**Fig. 8:**
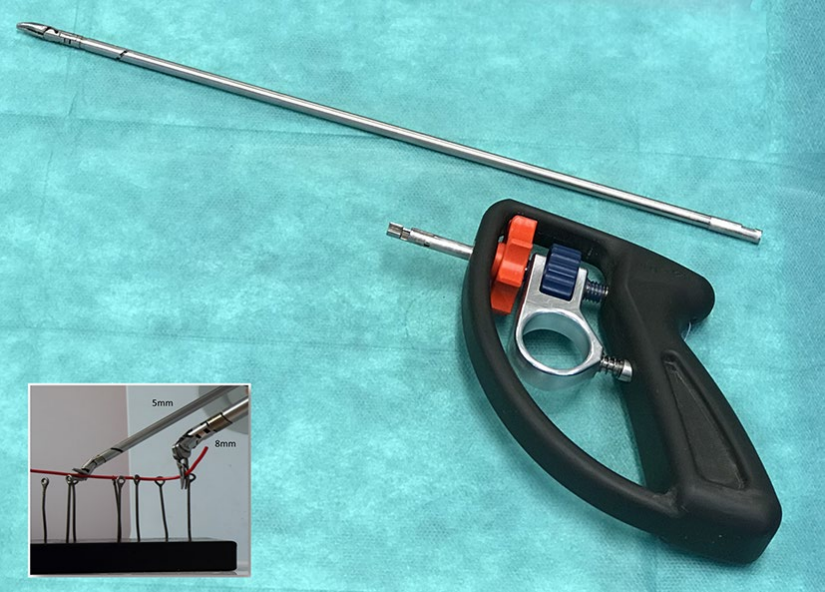
SATA grasper handle detached from shaft assembly. Window image: 5 mm and 8 mm SATA instruments in action during a wire chasing training task

Comparing the number of parts found in the shaft and tip of Intuitive Surgical’s EndoWrist with the SATA grasper of Fig. 8, a reduction of 17 components has been achieved. On the actuation side, moreover, approximately 20 further components normally used by the EndoWrist to guide and tension the actuation cable have been eliminated from the SATA grasper design [17].

For this study, 60 individual training exercises were conducted and the instrument was dismantled and reassembled at least 70 times. Inspection of the components after all the experiments showed no wear. This indicates that reusing the instrument for 30 procedures, as stated in the requirements, should be possible if a safety margin of two (i.e. 60 training trials/30 foreseen procedures) is respected. If a minimum of three SATA instruments are utilized per procedure (i.e. for cutting, manipulation, and closing) and each can be reused 30 times instead of ten, then in the potential situation that one in three laparoscopic surgeries (i.e. 7.5 million procedures annually) in 2025 is performed using articulating instruments [35, 36], a minimum of 1.5 million Instruments would be saved from disposal each following year.

### Limitations

Unlike in other designs, the relationship between pitch angle and tube rotation in the SATA grasper is not entirely constant due to the three-point hinge mechanism. Although this was never actually noticed during the user experiments, it should be considered when developing the control scheme for the use of the instruments in a robotic system. In the current SATA design, there are also sliding contact areas between the Nitinol flexors and metal components that can increase the actuation force if the instrument is fully articulated and loaded. Although none of the subjects reported any issues with it during the study, this articulation angle-dependent actuation force should be considered when this type of instrument is used in a robotic system. Finally, the assembly and disassembly tests were conducted by subjects with an engineering background at undergraduate level. Additional research is needed to determine whether the skills of CSSD employees can be compared with these students.

### Future work

Within the current state of development it remains unknown whether the presented instruments will ever be considered a disruptive technology. To show the advantage of this modular reusable steering technology over conventional steerable instruments, future steps should include a Health Tech Assessment (HTA) and clinical evaluation of the technology in developed and developmental area hospitals. Furthermore, cleaning validation should indicate the required knowledge/skills level of the cleaning and sterilisation department personnel that processes the instrument.

## Conclusion

By removing cables from the design, a fully functional, detachable, cableless 5.3 mm laparoscopic instrument was designed with two additional degrees of freedom at the tip. Apart from having an autoclavable handle, this instrument meets all design, functional, and user requirements set. It can be assembled and disassembled for cleaning and sterilization within an acceptable time frame and using low-tech cleaning methods. Box-trainer experiments showed that the additional degrees of freedom controlled by the handle interface are used efficiently.

## Electronic supplementary material

Below is the link to the electronic supplementary material.

Supplementary material 1 (MP4 92630 kb)
